# Stem Cells, Diet, and Physiology

**DOI:** 10.1093/geroni/igaa057.2642

**Published:** 2020-12-16

**Authors:** Daniela Drummond-Barbosa

**Affiliations:** Johns Hopkins Bloomberg School of Public Health, Baltimore, Maryland, United States

## Abstract

Nutrient availability, stresses, and aging affect tissue stem cells in multicellular organisms; yet, the underlying physiological mechanisms in vivo remains largely unexplored. Dr. Drummond-Barbosa pioneered using Drosophila to study the physiology of tissue stem cell regulation. Her laboratory played a major role in delineating how diet, brain insulin-like peptides, and the TOR nutrient sensor control the germline stem cell (GSC) lineage. They also discovered that adipocyte-specific disruption of amino acid transport, other nutrient signaling, and metabolic pathways causes distinct germline phenotypes. They also showed that nuclear receptors act in multiple tissues to affect the GSC lineage through direct and indirect mechanisms. More recently, her group has been exploring how other physiological stresses affect the GSC lineage. Her group’s studies point to extensive communication between the brain, adipocytes, hepatocyte-like cells, and the germline, and underscore the complexity of the physiological network that modulates stem cell lineage behavior.

